# A multilayer network model of neuron-astrocyte populations in vitro reveals mGluR_5_ inhibition is protective following traumatic injury

**DOI:** 10.1162/netn_a_00227

**Published:** 2022-06-01

**Authors:** Margaret E. Schroeder, Danielle S. Bassett, David F. Meaney

**Affiliations:** Department of Bioengineering, School of Engineering & Applied Science, University of Pennsylvania, Philadelphia, PA, USA; Department of Physics & Astronomy, College of Arts & Sciences, University of Pennsylvania, Philadelphia, PA, USA; Department of Electrical & Systems Engineering, School of Engineering & Applied Science, University of Pennsylvania, Philadelphia, PA, USA; Department of Neurology, Perelman School of Medicine, University of Pennsylvania, Philadelphia, PA, USA; Department of Psychiatry, Perelman School of Medicine, University of Pennsylvania, Philadelphia, PA, USA; Department of Neurosurgery, Perelman School of Medicine, University of Pennsylvania, Philadelphia, PA, USA

**Keywords:** Astrocyte, Traumatic injury, Multilayer network, Neuron-glia signaling

## Abstract

Astrocytes communicate bidirectionally with neurons, enhancing synaptic plasticity and promoting the synchronization of neuronal microcircuits. Despite recent advances in understanding neuron-astrocyte signaling, little is known about astrocytic modulation of neuronal activity at the population level, particularly in disease or following injury. We used high-speed calcium imaging of mixed cortical cultures in vitro to determine how population activity changes after disruption of glutamatergic signaling and mechanical injury. We constructed a multilayer network model of neuron-astrocyte connectivity, which captured distinct topology and response behavior from single-cell-type networks. mGluR_5_ inhibition decreased neuronal activity, but did not on its own disrupt functional connectivity or network topology. In contrast, injury increased the strength, clustering, and efficiency of neuronal but not astrocytic networks, an effect that was not observed in networks pretreated with mGluR_5_ inhibition. Comparison of spatial and functional connectivity revealed that functional connectivity is largely independent of spatial proximity at the microscale, but mechanical injury increased the spatial-functional correlation. Finally, we found that astrocyte segments of the same cell often belong to separate functional communities based on neuronal connectivity, suggesting that astrocyte segments function as independent entities. Our findings demonstrate the utility of multilayer network models for characterizing the multiscale connectivity of two distinct but functionally dependent cell populations.

## Author Summary

Astrocytes communicate bidirectionally with neurons, enhancing synaptic plasticity and promoting the synchronization of neuronal microcircuits. We constructed a multilayer network model of neuron-astrocyte connectivity based on calcium activity in mixed cortical cultures, and used this model to evaluate the effect of glutamatergic inhibition and mechanical injury on network topology. We found that injury increased the strength, clustering, and efficiency of neuronal but not astrocytic networks, an effect that was not observed in injured networks pretreated with a glutamate receptor antagonist. Our findings demonstrate the utility of multilayer network models for characterizing the multiscale connectivity of two distinct but functionally dependent cell populations.

## INTRODUCTION

Astrocytes, the most abundant glial cell in the brain, can modulate the activity of neurons through the uptake of neurotransmitters and release of gliotransmitters (Di Castro et al., 2011; J. Kang, Jiang, Goldman, & Nedergaard, 1998; Panatier et al., 2011; Perea & Araque, 2007; J. T. Porter & McCarthy, 1996). One such pathway is uptake of synaptic glutamate through the astrocytic metabotropic glutamatergic receptor subtype 5 (mGluR_5_) (Panatier & Robitaille, 2016), Figure 1. In turn, glutamate release from neurons may trigger the subsequent release of glutamate and other neurotransmitters from astrocytes, a process commonly termed gliotransmission. Through gliotransmission, astrocytes can enhance or depress synaptic plasticity and the synchronization of neuronal circuits (Pascual et al., 2005; Poskanzer & Yuste, 2011; Serrano, Haddjeri, Lacaille, & Robitaille, 2006). For neurons, the activation of mGluR_5_ is associated with both strengthening and weakening synapses (Gladding, Fitzjohn, & Molnár, 2009) and playing a role in some neuropsychiatric disorders (Bear, Huber, & Warren, 2004; Dölen et al., 2007). Although prior studies disagree on the relative abundance of mGluR_5_ in neurons versus astrocytes (Kettenmann & Schachner, 1985; Saunders et al., 2018; Y. Zhang et al., 2014), astrocyte mGluR_5_ has been identified as one of the most prominent receptors implicated in astrocytic detection of neurotransmitters, Ca2+ signaling, and downstream synaptic modulation (Panatier & Robitaille, 2016; Panatier et al., 2011; J. T. Porter & McCarthy, 1996; X. Wang et al., 2006). Despite recent advances in understanding neuron-astrocyte signaling, comparatively little is known about astrocytic modulation of neuronal population activity via mGluR_5_ signaling, particularly in cases where that activity has been altered by disease and by injury. In this study, we sought to define the role of astrocytes in modulating neural circuits before and after targeted neuronal injury and evaluate how mGluR_5_ signaling shapes the functional connections among, and across, these two distinct cell populations in a living neural microcircuit.

**Figure 1.**
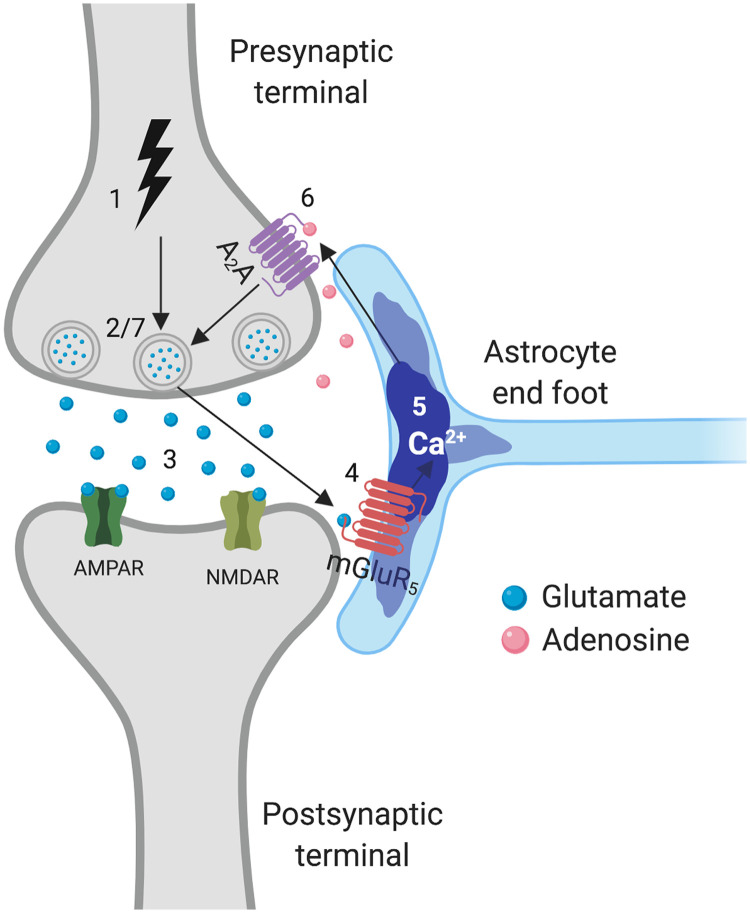
The tripartite synapse mediated through mGluR_5_. An action potential arrives at the presynaptic terminal (1) and triggers glutamate release (2–3). Glutamate acts on astrocytic mGluR_5_ (4), leading to an increase in astrocyte Ca^2+^ (5). The astrocyte releases adenosine (6), which is taken up by presynaptic A_2_A receptors, and alters the probability of neurotransmitter release (7). Created with BioRender.

Based on the large body of evidence supporting a role for astrocytes in establishing neural population synchronization and in protecting against neural degeneration following traumatic brain injury (TBI) (Burda, Bernstein, & Sofroniew, 2016; Myer, Gurkoff, Lee, Hovda, & Sofroniew, 2006), astrocytes may play a key role in recovering neuronal network synchrony, which is disrupted following moderate mechanical injury to neural tissue (W. H. Kang et al., 2015; Patel, Ventre, & Meaney, 2012). Previous studies examining astrocyte response to mechanical stretch injury in vitro directly triggered injury cascades in astrocytes (Charles, Merrill, Dirksen, & Sandersont, 1991; Ellis, McKinney, Willoughby, Liang, & Povlishock, 1995; Rzigalinski, Weber, Willoughby, & Ellis, 1998) and reactive gliosis (Miller et al., 2009). Mediated by intracellular calcium waves in astrocytes that originate from sites of mechanical injury, past work shows signaling from astrocytes can temporarily silence neuronal network after trauma (Choo et al., 2013). In addition, therapeutically manipulating the receptors that control the extent of astrocyte intercellular waves after trauma also lead to an improvement in outcome after injury. However, the interdependence of targeted neuronal injury and astrocyte activity, and how these cell types interact to recover activity in neuronal activity in networks, is not known. Determining more precisely the nature of neuron-astrocyte communication following injury may reveal mechanisms on how neural circuits recover from trauma, and the role that surrounding astrocyte networks play in this recovery process.

In vivo, the stress transferred to the cellular and subcellular components is complex and heterogeneous (Bain, Shreiber, & Meaney, 2003; Bar-Kochba, Scimone, Estrada, & Franck, 2016; Cloots, Van Dommelen, Nyberg, Kleiven, & Geers, 2011; LaPlaca, Cullen, McLoughlin, & Cargill II, 2005; Pan, Sullivan, Shreiber, & Pelegri, 2013). Rather than applying a uniform membrane deformation to a mixture of integrated neuronal and glial cells in a network, we sought to mimic the heterogeneous nature of mechanical trauma by using a localized injury model developed recently (Mott, von Reyn, Firestein, & Meaney, 2021). One of the primary events that occurs instantly during trauma is the activation of receptors and channels in the membrane (Iwata et al., 2004; Patel, Ventre, Geddes-Klein, Singh, & Meaney, 2014), the instantaneous release of neurotransmitters and gliotransmitters (Choo et al., 2013; LaPlaca & Thibault, 1998), and the appearance of nonspecific pores in the plasma membrane (Farkas, Lifshitz, & Povlishock, 2006; Kilinc, Gallo, & Barbee, 2009; LaPlaca et al., 2019; Tehse & Taghibiglou, 2019) that can impair neuronal and glial function. Within this injury model, we chose to focus on the relative role of mGluR_5_ receptors after traumatic neuronal injury in vitro. The mGluR_5_ receptor is an appealing receptor target because it is expressed by both neurons and astrocytes, yet very little is known about its effect on network activity and structure. Moreover, evidence points to an uncertain role for mGluR_5_ on neuronal outcome. Some studies show that activation of mGluR_5_ improves outcome following injury (Chen et al., 2012a; Loane, Stoica, Byrnes, Jeong, & Faden, 2013; Loane et al., 2014), while other reports show inhibition is necessary to reduce neuronal degeneration and improve functional recovery (Chen et al., 2012b; Movsesyan et al., 2001). Part of this controversy could be that these studies could not study how the activation of these receptors affects the structure and topology of neuronal microcircuits after trauma. With the aforementioned injury mechanisms occurring at the moment of injury, our approach to determine the mechanistic role of mGluR_5_ on neuronal and astrocyte network structure required us to block mGluR_5_ prior to mechanical trauma.

Past work to study the dynamics between astrocytes and neurons often relies on in situ techniques (e.g., acute slice preparations) or transgenic approaches, neither of which allows an exact determination of how neighboring astrocytes influence the activity of neurons, or vice versa. Although the emergence of probes to record cellular-level resolution of neuronal networks in vitro and in vivo emerged in the past decade (Akerboom et al., 2012; Tian et al., 2009), the relationship between the timing and influence of astrocytic activation—which often occurs over several seconds—on the subsequent neuronal activity patterns by using these probes or other technologies remains largely unknown. One key obstacle is that this relationship depends on both proximity of one cell type to another, the resulting connections each cell types forms within a larger network, and the complex timing of these interactions.

The emerging field of network neuroscience, which uses tools from graph theory to analyze neural communication as reflected in the topology of interaction patterns, provides an apt set of tools for analyzing multicellular activity and, in particular, how this activity can be causally linked across cell populations, or layers. Multilayer networks are a generalized framework for incorporating multiple types of relations between participating units, capable of describing and modeling a wide range of complex systems (Betzel & Bassett, 2017; Kivelä et al., 2014; Muldoon & Bassett, 2016; Vaiana & Muldoon, 2020). The network formed from bidirectional neuron-astrocyte functional connectivity lends itself to a multilayer network model, where astrocytes and neurons are encoded as nodes in a multilayer network. In analyzing dynamic activity occurring across both neurons and astrocytes within the framework of a single network, one can objectively assess the relative influence of one cell type on another, understand whether these cell layers communicate with each other, and identify the role of exogenous factors, such as mGluR_5_ signaling and traumatic injury, on network structure and function.

The objective of this study was threefold: to determine the spatial and functional topology of neuron-astrocyte networks using a multilayer network model, to characterize changes in topology resulting from single-cell injury and mGluR_5_ inhibition, and to determine whether fully functional cellular communication is required for postinjury recovery of baseline network topology. We show that targeted neuronal injury, but not pharmacological blockade of glutamatergic signaling via mGluR_5_, altered higher order topological properties of multilayer networks, including clustering and global efficiency of the neuronal layer. Our results also indicate that mGluR_5_ inhibition modulates the topological impact of injury. More broadly, our findings demonstrate the utility of multilayer network models for characterizing the micro-, meso-, and global-scale connectivity of two morphologically and genetically distinct but functionally dependent cell populations.

## RESULTS

**Figure 2.**
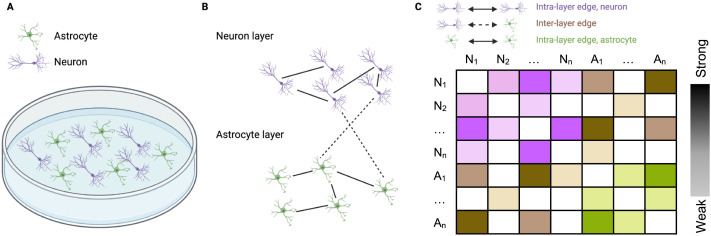
A multilayer network model of neurons and astrocytes in co-culture. (A) Simplified schematic of neurons (purple) and astrocytes (green) in co-culture. Morphology and size are exaggerated. (B) Cells are separated into two distinct layers based on their identity. Layers are made up of exclusively one cell type. Each cell may be connected to cells in its own layer or to cells in the other layer. Intralayer edges are depicted with full lines and interlayer edges with dashed lines. (C) Example adjacency matrix for the multilayer network shown in panel B. The purple and green weights correspond to intralayer edges and the brown weights correspond to interlayer edges. Color saturation indications strength of connection.

We used a multilayer and two single-layer network models to characterize population-level neuron and astrocyte segment dynamics. Neural cell cultures can be modeled as networks without violating the foundational modeling assumptions of network science (Bassett, Zurn, & Gold, 2018). A node represents a single neuron in the neural network, and it represents an astrocyte process in the astrocyte network. We use moderate speed (>10 frames per second) imaging of intracellular calcium in individual cells to detect transient events in these network nodes. In neurons, these transient changes in fluorescence represent one or more action potential event(s) (Akerboom et al., 2012; Tian et al., 2009), which can release neurotransmitters that can activate surface receptors on astrocytes to subsequently release of calcium from intracellular stores (Araque, Parpura, Sanzgiri, & Haydon, 1999). Likewise, calcium transients in astrocytes can release gliotransmitters which influence neuronal activity (Araque, Parpura, Sanzgiri, & Haydon, 1998; Parpura & Haydon, 2000). Therefore, tracking the timing of fluorescent changes in each cell type provides a rich dataset containing the spatial location and temporal activity of each cell in the population. Edges are determined by functional connectivity between nodes based on fluorescence activity over time (Patel, Man, Firestein, & Meaney, 2015). Each node was assigned to the neuron or astrocyte layer depending on its identity (Figure 2, Supporting Information Section B). Each edge representing a functional connection between an astrocyte and a neuron, or between two astrocytes or two neurons, was weighted by the functional connectivity between them (see Methods, Supporting Information Section C).

Only cells that were inactive across all conditions were eliminated from the graph, allowing some nodes to have no edges in certain conditions. Dishes were analyzed as separate networks, each with a variable number of nodes (Supporting Information Figure S2C). Many useful statistics can be calculated from these graphs, the simplest being the number of nodes and edges. More complex network parameters provide additional information about how cells are functionally connected. Because the plating density of neurons and astrocytes varies between dishes and between isolations, we calculated network measures that can be normalized for network size (Supporting Information Section D). Mean degree, density, nodal strength (normalized to network size), clustering coefficient, betweenness centrality (also normalized), and global efficiency were calculated for each network and for a random null network model in which both degree distribution and density were preserved. The rationale for reporting these metrics was biologically driven, as they capture general properties of multicellular communication: its overall density, the extent to which connected cells signal to shared neighbors, and the extent to which cells act as hubs, facilitating signaling between other cells. A random, nondistance-dependent null model was chosen because the attachment of cortical neurons and astrocytes to the dish upon plating was random, as was their wiring to other cells after plating.

### mGluR_5_ Localizes to Neurons and Astrocytes in Culture

To verify the presence and assess the relative abundance of mGluR_5_ on neurons and astrocytes in our cultures, we stained for mGluR_5_; microtubule associated protein 2 (MAP2), a neuronal marker; glial fibrillary acidic protein (GFAP), an astrocytic marker; and nuclei (Hoechst stain), for counting purposes. Six dishes of näıve cells were fixed at DIV 10, the same age at which experimental cells were treated and imaged, stained using immunofluorescent antibodies (Supporting Information Section G), and imaged in three fields of view. MAP2 and mGluR_5_ co-localized in 99% of neurons (*n* = 6 dishes, *n* = 3 fields of view each, *n* = 525 neurons total), verifying expression of the receptor on neurons. GFAP and mGluR_5_ co-localized in 76% of astrocytes (*n* = 6 dishes, *n* = 3 fields of view each, *n* = 119 astrocytes total), confirming the expression of the receptor in astrocytes. However, anti-mGluR_5_ intensity was visibly lower in astrocytes than in neurons (Supporting Information Figure S1), suggesting that neurons in our cultures express more mGluR_5_ than astrocytes. A notable limitation of GFAP as an astrocyte marker is that it does not stain fine astrocyte processes or microdomains (Shigetomi et al., 2013), where mGluR_5_ is more likely to be located (Panatier & Robitaille, 2016; Panatier et al., 2011). Thus, our stain may not have captured the full extent of astrocytic mGluR_5_ expression.

### Blockade of Glutamatergic Signaling and Mechanical Injury Decrease the Activity of Neurons, but Not Astrocytes

As a first-order measure of network activity, we calculated the mean frequency of calcium events for each cell type in each dish (Supporting Information Section A, Section B), and then averaged these values over all cells in the field of view that were active at any time point during the experiment. Neuronal event rate decreased following treatment with 2-Methyl-6-(phenylethynyl)pyridine (MPEP), a potent mGluR_5_ inhibitor, immediately after drug addition but before injury. Following injury, neuronal event rate was lower in the MPEP-treated and injured arms relative to untreated control dishes (Supporting Information Figure S2A). However, there was no significant difference in event rate between baseline and postinjury time points for any of the four groups. In the untreated, uninjured control group (MEM, Sham) and the MPEP-treated uninjured group, there was a decrease in event rate following drug treatment, followed by a subsequent increase to near-baseline levels or higher at the injury time point. This suggests that the addition of vehicle depresses event rate, which is subsequently recovered in uninjured networks. Both mGluR_5_ blockade and neuronal injury significantly reduced neuronal event rate relative to untreated dishes (Supporting Information Figure S2A and Table S1; Tukey’s multiple comparisons test following 2-way ANOVA, at postinjury time point, *p* = 0.0111 for MEM, Sham vs. MEM, Injury, *p* = 0.0145 for MEM, Sham vs. MPEP, Sham, *p* = 0.0004 for MEM, Sham vs. MPEP, Injury). Astrocytic event rate was unchanged following neuronal injury and/or mGluR_5_ inhibition (Supporting Information Figure S2B, Table S1). These results suggest that any changes in functional network topology may be mediated directly through decreased activity in neural layers, while changes in astrocyte layer topology are unlikely to be mediated through decreased astrocyte activity.

### Nonrandom Topology of Neuron, Astrocyte, and Multilayer Networks

**Figure 3.**
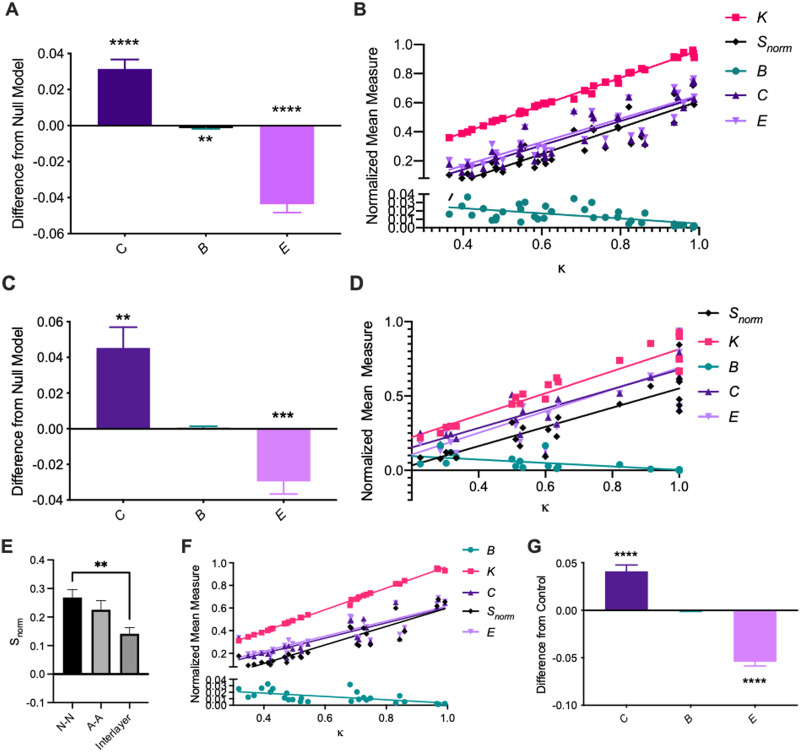
Characterization of neuron-neuron, astrocyte-astrocyte, and multilayer neuron-astrocyte functional network topology (A) Difference from random null model of calculated mean clustering coefficient *C*, normalized betweenness centrality *B*, and global efficiency *E* for neuron-neuron networks at baseline. We observe significantly larger clustering coefficients and significantly lower global efficiency than expected from a random null model. (B) Mean clustering coefficient *C*, normalized betweenness centrality *B*, normalized degree *K*, normalized strength *S*_*norm*_, and global efficiency *E* vs. mean density *κ* for each dish at the third imaging time point (1 hour post-injury), pooled across conditions. We observe clear positive correlations as assessed by a linear regression for *K*, *S*_*norm*_, *C*, and *E*, and a clear negative correlation for *B*. (C) Same as A, for astrocyte-astrocyte networks. A similar network topology is observed. (D) Same as B, for astrocyte-astrocyte networks. (E) Mean normalized strength of neuron layer, astrocyte layer, and interlayer connections. (F) Same as B, for multilayer networks. (G) Same as A, for multilayer networks. Error bars indicate standard error of the mean (SEM) and asterisks indicate statistical significance (*p ≤ 0.05, **p ≤ 0.01, ***p ≤ 0.001, ****p ≤ 0.0001).

Next, we sought to characterize functional network topology, which is a higher order measure of coherent population-level activity. Weighted adjacency matrices were generated by calculating the Pearson’s correlation coefficient between pairs of filtered and scaled astrocyte calcium traces (Supporting Information Section C). In addition to the network density *κ*, the global efficiency *E*, the clustering coefficient *C*, the normalized degree *K*, and the normalized betweenness centrality *B*, we calculated the mean normalized nodal strength *S* of each network and normalized it based on the number of nodes in the network. To test whether these network statistics were topologically meaningful (nonrandom) after controlling for network size and density, we compared them to those of a null model in which connections were randomly rewired while preserving the degree distribution and density. Relative to randomized networks, neuron networks exhibited significantly greater clustering coefficient (Figure 3A; one-sample *t* test on differences between actual values and null model values of *C*, *t* = 6.010, *df* = 35, *p* < 0.0001), significantly lower betweenness centrality (one-sample *t* test on differences between actual values and null model values of *B*, *t* = 2.839, *df* = 35, *p* = 0.0075) and significantly lower global efficiency (one-sample *t* test on differences between actual values and null model values of *E*, *t* = 9.518, *df* = 35, *p* < 0.0001). That is, neuron networks have more interconnected triads and fewer long-distance connections, which promote efficiency of communication between nodes, than would be expected based purely on their density. As would be expected for random networks of the same size and degree distribution, the clustering coefficient *C* and global efficiency *E* were strongly correlated with edge density, while betweenness centrality *B* was anti-correlated (Figure 3B; see Supporting Information Table S2 for statistics). The degree to which these topological measures depended on density in our observed networks was slightly different than that of randomized control networks (Supporting Information Figure S3, Table S2).

In general, astrocyte networks were small and sparse. After eliminating segments that were not active at any time during the experiment, only 28 out of 36 dishes remained, with a median of about 3 active whole cells and about 15 active astrocyte processes per dish (Supporting Information Figure S2C, Figure S4D). The number of whole astrocytes correlated well with the number of active astrocyte segments, with each active cell having on average around five active segments (Supporting Information Figure S4D). Similar to the neuron networks, functional astrocyte networks displayed significantly higher clustering coefficient (one-sample *t* test on differences between the actual values and null model values of *C*, *t* = 3.887, *df* = 21, *p* = 0.0009) and significantly lower global efficiency (one-sample *t* test on differences between actual values and null model values of *E*, *t* = 4.167, *df* = 21, *p* = 0.0004) than control networks (Figure 3C). For astrocyte networks, *B* was not significantly different from that of randomized control networks (one-sample *t* test on differences between actual values and null model values of *B*, *t* = 0.6922, *df* = 21, *p* = 0.4964). As for neuron networks, *C* and *E* were strongly correlated with edge density for astrocyte networks, while betweenness centrality *B* was anticorrelated (Figure 3D; see Supporting Information Table S2 for statistics). Again, the degree to which these topological measures depended on density in our observed networks was slightly different than that of randomized control networks (Supporting Information Figure S3, Table S2).

While it is useful to consider neuron and astrocyte networks separately, neurons and astrocytes do not exist in isolation. To better understand the interactions between neurons and astrocytes, and to assess whether the topology of these subpopulations is independent of or informed by the other, we modeled neuron-astrocyte functional interactions as a multilayer network (Figure 2). Edges for multilayer adjacency matrices were assigned weights from the functional connectivity between neurons and neurons, between astrocytes and astrocytes, and between neurons and astrocytes, as measured by the Pearson’s correlation coefficient of filtered and scaled calcium traces (Supporting Information Section C). Strength and density were calculated for the entire multilayer network, for neuron and astrocyte layers separately, and for interlayer connections separately. Mean normalized betweenness centrality, mean clustering coefficient, and global efficiency were calculated for the entire multilayer network and for control networks generated as previously described. Random control networks were generated by randomizing the entire multilayer adacency matrix, rather than A-A and N-N layers separately.

We found that intralayer connections are stronger than interlayer connections (Figure 3E), with mean nodal strength being highest in the neuron layer and lowest in interlayer connections (Tukey’s multiple comparisons test following one-way ANOVA for N-N vs. interlayer connections, *q* = 4.631, *p* = 0.0046, *df* = 70). Consistent with our findings for neuron and astrocyte networks individually, multilayer networks exhibit significantly larger clustering coefficient *C* (one-sample *t* test on differences between actual values and null model values, *t* = 6.190, *df* = 26, *p* < 0.0001) and lower global efficiency *E* (one-sample *t* test on differences between actual and null, *t* = 12.27, *df* = 26, *p* < 0.0001) than their randomized counterparts (Figure 3G). No differences in betweenness centrality *B* were observed (one-sample *t* test on differences between actual and null, *t* = 1.095, *df* = 26, *p* = 0.2835). Also consistent with our findings for the individual layers, *C* and *E* were strongly correlated with edge density for multilayer networks, while betweenness centrality *B* was anticorrelated (Figure 3F, Supporting Information Table S2). Again, the degree to which these topological measures depended on density in our observed networks was slightly different than that of randomized control networks (Supporting Information Figure S3, Table S2).

### Mechanical Injury Alters Neuron and Multilayer, but Not Astrocyte Layer Network Topology

Because glutamate is an important neural and glial transmitter, we targeted mGluR_5_ to inhibit neuron-to-neuron (N-N) and neuron-to-astrocyte (N-A) communication. We then measured the resulting changes in network topology by calculating the mean nodal strength (*S*), density (*κ*), clustering coefficiency (*C*), betweenness centrality (*B*), and global efficiency (*E*) of functional neuronal networks after disruption of mGluR_5_-mediated N-N and N-A signaling (Supporting Information Section D). Importantly, for both neurons and astrocytes, mean nodal strength and edge density were not positively correlated with mean event rate (Supporting Information Figure S5A, Figure S6A). Thus, any observed changes in network density and/or topology from our manipulations are likely not caused by spurious changes in calcium trace correlation due to reduced or increased activity.

Both administration of the mGluR_5_ antagonist and neuronal injury led to a modest increase in mean edge density and mean nodal strength at the final experimental time point, though this different was not statistically significant (Supporting Information Figure S5B–D, one-way ANOVA between treatment groups for *S*_*norm*_ 1 hour post-injury, *F*(3, 32) = 1.741, *p* = 0.1784). To assess the impact of mGluR_5_ inhibition, injury, and their interaction on multilayer network topology, we fit a generalized linear model to multilayer graph statistics including the betweenness centrality *B*, the clustering coefficient *C*, and the global efficiency *E*. The outcome variable of interest was calculated for each dish at the third time point and regressed on its group assignment while controlling for mean nodal strength as a covariate of noninterest. We found that mean nodal strength was the greatest, and most statistically significant, covariate of all measures (Supporting Information Table S3). The dependence of these topological properties on density is also evidenced by a strong correlation between the density and the clustering coefficient *C*, the global efficiency *E*, and the betweenness centrality *B* (Figure 3B). At the final experimental time point, mean nodal strength *S* of multilayer networks was significantly altered by injury only (GLM with *S* as outcome variable and MPEP, Injury, MPEP + Injury interaction term, and event rate as covariates; two-sided *z* test on estimated coefficient for *S* on Sham dummy variable, *β* = −0.2046, *z* = −2.183, *p* = 0.029). After controlling for mean nodal strength, the GLM revealed that injury increased clustering coefficient *C* and global efficiency *E* of neuronal networks (two-sided *z* test on estimated coefficient for *C* on sham dummy variable, *β* = −0.0346, *z* = −2.450, *df* = 31, *p* = 0.014; coefficient on sham dummy variable for *E*, *β* = −0.0224, *z* = −2.207, *df* = 31, *p* = 0.027). This effect was not seen in the MPEP + Injury group, suggesting that the addition of MPEP attenuates the effect of injury.

We observed no significant difference in density or mean strength of astrocyte networks after treatment with anti-mGluR_5_ or after injury (Supporting Information Figure S6B–D). As for neuron networks, we used a GLM and regressed out mean nodal strength in astrocyte networks to isolate the effect of our manipulations. For all measures, there was no significant effect of MPEP administration, injury, or their interaction (Supporting Information Table S4). Furthermore, there was no significant effect of MPEP, injury, or their interaction on mean nodal strength of astrocyte networks at the final time point (GLM with *S* as outcome variable and MPEP, Injury, MPEP + Injury interaction term, and event rate as covariates, all *p* values from *z* tests on coefficients above 0.05).

Finally, as for the neuron and astrocyte layers, we fit a GLM to isolate the effect of our manipulations on full multilayer topology at the final experimental time point (1 hour postinjury). Unlike the behavior of the neuronal layer, there was no significant effect of MPEP, injury, or their interaction on either mean nodal strength of multilayer networks or interlayer connections at the final time point (GLM with *S* as outcome variable and MPEP, Injury, MPEP + Injury interaction term, and event rate as covariates). However, in the injury-only group, but not the MPEP + Injury group, we observed a significant increase in the clustering coefficient *C* (two-sided *z* test on estimated coefficient on Sham dummy variable for *C*, *β* = −0.0368, *z* = −2.473, *df* = 22, *p* = 0.013), but no change in the global efficiency *E* or betweenness centrality *B*. Neither *C*, *B*, or *E* were significantly different from control in MPEP-treated or MPEP-treated and injured groups, suggesting that the effect of injury on multilayer network topology is attenuated by mGluR_5_ inhibition. It is notable that injury had a similar effect in direction and magnitude on *C* for neuronal and multilayer networks, but that the observed increase in *E* for injured neuronal networks was not observed in multilayer networks.

To measure the impact of the relative abundance of neurons to astrocytes on multilayer topology, we subsampled the neuron population to equal the number of astrocytes and recalculated network statistics on these synthetic half-neuron, half-astrocyte networks (Supporting Information Section E). Subsampled multilayer networks exhibited similar baseline topology (Supporting Information Figure S7), but slightly different response behavior (Supporting Information Table S7) than full multilayer networks, suggesting that the relative abundance of neurons partially drives changes in topology.

### Mechanical Injury Increases the Dependence of Functional Connectivity on Spatial Proximity in Mesoscale Multilayer Networks

**Figure 4.**
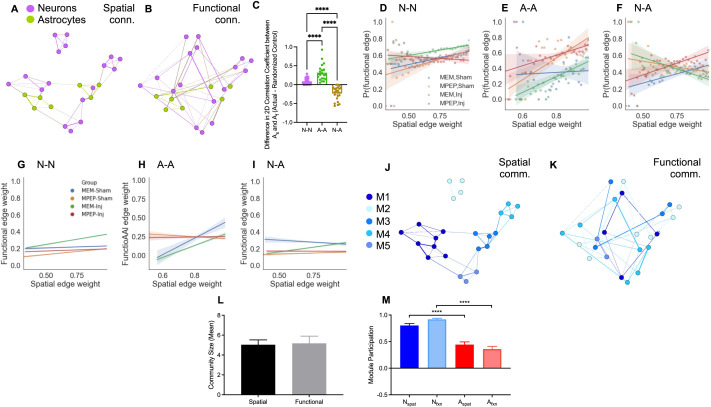
Spatial-functional dependency in multilayer networks. (A) A representative graph of a multilayer network with weighted edges based on spatial proximity. (B) A representative graph of a multilayer network with weighted edges based on functional connectivity. (C) Difference in 2-D correlation coefficient between actual and random functional adjacency matrices and spatial adjacency matrices for neuron-neuron (purple), astrocyte-astrocyte (green), and neuron- astrocyte (brown) multilayer network layers. (D) Functional connection probability (see Methods) versus spatial edge weight for neuron-neuron connections at the third experimental time point for each experimental group. Shading indicates 95% confidence interval on the slope (Supporting Information Table S8, *n* = 30 bins from 16,653 edges total). (E) Same as D for astrocyte-astrocyte connections (*n* = 25–29 bins from 2,271 edges total). (F) Same as D, E for neuron-astrocyte connections (*n* = 26–28 bins from 10,437 edges). (G–I) Functional edge weight versus spatial edge weight for neuron-neuron (G), astrocyte-astrocyte (H), and neuron-astrocyte (I) layers of the multilayer network. Shown is the best-fit line from simple linear regression for the four experimental groups at the final time point (1 hour post-injury). Shading indicates 95% confidence interval on the slope (Supporting Information Table S9). (J) Community structure of the network shown in panel A with modules determined based on spatial proximity. Nodes of the same color belong to the same spatial module. (K) Community structure of the network shown in panel J with modules determined based on functional connectivity. Nodes of the same color belong to the same functional module. If functional connectivity were based on spatial proximity, the modules in panels J and K would be the same or highly similar. (L) Mean community size does not differ significantly between functional and spatial multilayer communities, as the spatial tuning parameter was adjusted to minimize this difference. (M) Average module participation, the fraction of modules that contain at least one of that cell type, as determined based on spatial distance and functional connectivity for both neurons and astrocytes. Error bars indicate standard error of the mean (SEM) and asterisks indicate statistical significance (*p ≤ 0.05, **p ≤ 0.01, ***p ≤ 0.001, ****p ≤ 0.0001).

In addition to functional connectivity, spatial distance between nodes might shape network topology (Figure 4A–B, Supporting Information Figure S8A–D), though the baseline magnitude of this dependence is likely to be small for out networks, given the spatially random plating of cells in dissociated culture and the scale of the imaging field of view (usually less than 100 cells per field of view). The location of each cell in 2D space was extracted from calcium fluorescence recordings. Spatial adjacency matrices were generated such that nodes with the shortest radial distance were most strongly connected. Spatial connectivity strength could influence functional connectivity in several ways, including (1) the probability of a functional connection existing at all and/or (2) the strength of nonzero functional connections. We examined both of these by regressing functional edge probability and functional edge weight on spatial edge weight for multilayer networks of each experimental condition at the final experimental timepoint.

To investigate whether connection probability depends on spatial connectivity, we grouped functional edges into 30 bins based on their spatial connection weight and calculated connection probability as the number of nonzero edges in the bin divided by the total number of edges in the bin. We then regressed functional connection probability against the mean spatial connection weight in the bin (Figure 4D–F). For the neuronal layer, we found a positive correlation between spatial edge weight and functional connection probability for all conditions except MPEP, Injury (Supporting Information Table S8A). While the slope of the best-fit line was similar for MEM/Sham MPEP/Sham, and MEM/Inj groups at the final time point, networks subjected to injury only had a larger functional connection probability for a given spatial connection weight (simple linear regression, *R*^2^ = 0.377, *p* = 0.0003 for MEM, Inj). There was no significant linear relationship between functional connection probability and spatial connection weight for MPEP-treated injured dishes (simple linear regression, 95% CI of slope [−0.214, 0.029], *p* = 0.013).

For the astrocyte layer, there was no significant linear relationship between functional connection probability and spatial connection weight (Figure 4E simple linear regression, 95% CI of slope [−0.755, 0.990], *p* = 0.7840). MPEP treatment, injury, and the combination of the two resulted in a significant positive correlation between functional connection probability and spatial connectivity (Supporting Information Table S8B). For interlayer connections, we found a positive correlation between spatial edge weight and functional connection probability for untreated dishes (Figure 4F, simple linear regression, 95% CI of slope [0.179, 0.70], *p* = 0.00187). Both injury and MEM lessened this dependency (Supporting Information Table S8C). Interestingly, MPEP-treated, injured dishes had a slope closer to baseline (simple linear regression, 95% CI of slope [0.319, 0.801], *p* = 0.0001), suggesting a potential protective effect of MPEP treatment on distance-dependent interlayer connections against injury. Taken together, these results suggest complex, differential effects of injury and mGluR_5_ inhibition and mechanical injury on the functional edge probability and functional edge strength for intra- and interlayer connections, which are discussed further below.

To test whether the strength of functional connections depends on spatial proximity in our networks, we regressed the the edge weights in the functional adjacency matrix versus the edge weight for the same node pair in the spatial adjacency matrix for all connection types in our multilayer networks (neuron-neuron, neuron-astrocyte, and astrocyte-astrocyte) at baseline. We found a very small but significant correlation between spatial and functional weights for neuron-neuron and astrocyte-astrocyte layers (simple linear regression; neuron-neuron, 95% CI of slope [0.1463, 0.1823], *R*^2^ = 0.006624, *df* = 48,140, *p* < 0.0001; astrocyte-astrocyte, 95% CI of slope [0.2373, 0.3816], *R*^2^ = 0.01501, *df* = 4,643, *p* < 0.0001; neuron-astrocyte, 95% CI of slope [−0.2602, 0.01779], *df* = 20,733, *p* = 0.7127). When measured as a 2-dimensional correlation coefficient (Figure 4C, the spatial-functional agreement was significantly stronger than for random networks for neuron-neuron and astrocyte-astrocyte connections (95% CI of mean difference for N-N connections, [0.07024, 0.1060]; 95% CI of mean difference for A-A connections, [0.2166, 0.4144]) but weaker for interlayer neuron-astrocyte connections (95% CI of mean difference for N-A connections, [−0.3153, −0.1516]).

If the strength of functional neuronal connections are not spatially restricted, as the observed low correlation between spatial and functional edge weight would suggest, neurons and astrocyte microdomains must be able to form relatively strong long-distance functional connections. Assuming there is both a spatial (albeit small) and nonspatial component influencing functional connectivity, and that the functional, but not spatial, component is disrupted following injury, then mesoscale topology should change to favor connectivity governed by short-distance spatial connections following injury. We note that these short-distance spatial connections could be long-distance topological connections. To test this hypothesis, we compared the correlation between spatial and functional connectivity weights in the functional or spatial adjacency matrix at the final experimental time point between different experimental groups for the neuron-neuron, astrocyte-astrocyte, and interlayer connections (Figure 4). We found that injury increased the correlation between spatial and functional connectivity for the neuronal layer (Figure 4G, simple linear regression, *R*^2^ = 0.0 for MEM, Sham, *R*^2^ = 0.01 for MEM, Inj), astrocyte later (Figure 4H, simple linear regression, *R*^2^ = 0.075 for MEM, Sham, *R*^2^ = 0.096 for MEM, Inj), and interlayer connections (Figure 4I, simple linear regression, *R*^2^ = 0.001 for MEM, Sham, *R*^2^ = 0.0007 for MEM, Inj; Supporting Information Table S9). Treatment with MPEP decreased the spatial-functional edge weight correlation for the astrocyte layer (simple linear regression, *R*^2^ = 0.075 for MEM, Sham, *R*^2^ = 0.002 for MPEP, Sham), but increased it for the neuronal layer (simple linear regression, *R*^2^ = 0.0 for MEM, Sham, *R*^2^ = 0.006 for MPEP, Sham), while it did not affect interlayer spatial-functional correlation (Supporting Information Table S9). As we observed for neuronal and multilayer topology, pretreatment of injured dishes with MPEP attenuated the full effect of injury for the neuronal layer (simple linear regression, *R*^2^ = 0.006 for MEM, Inj, *R*^2^ = 0.001 for MPEP, Inj) and interlayer connections (simple linear regression, *R*^2^ = 0.007 for MEM, Inj, *R*^2^ = 0.0 for MPEP, Inj) Together, these results indicate that injury triggers a process of forming more local spatial networks, implying a loss in the coordination of signaling across physical networks, that is influenced by mGluR_5_ signaling.

### Modularity Analysis Reveals Community Association of Neurons and Astrocytes is Not Determined by Spatial Proximity at the Mesoscale

In addition to local and global topological statistics, we sought to understand the community structure of multilayer networks, which can be used to study dynamics in a subset of a larger network. Further, we asked whether community structure depends on the physical distance between nodes. To do so, we compared the modularity predicted by functional and spatial connectivity. The modularity of both functional and spatial graphs was determined by maximizing the quality function (Supporting Information Equation S13), constructed with a Newman-Girvan null model (Newman & Girvan, 2004) (Supporting Information Figure S13), using a Louvain-like locally greedy algorithm implemented in MATLAB (Jutla, Jeub, & Mucha, 2011). The spatial tuning parameter was adjusted to minimize the difference between the number of communities in the spatial networks and the number of communities in the functional networks. To compare the modularity predicted by spatial and functional graphs, we calculated the Adjusted Rand Index (*ARI*; Rand, 1971) between the partitions of spatial and functional networks into communities (Supporting Information Equation S17). The Rand Index is equal to one when two community partitions are exactly equal, and zero when there is no overlap. The *ARI* adjusts for the probability of two cells being assigned to the same module by chance, and can take on negative values.

Despite a small but significant correlation between functional and spatial connectivity, we found that community structure differed between functional and spatial networks for neuron-neuron networks (Supporting Information Figure S8E–F), with the *ARI* between functional and spatial partitions being no different from zero (neuron networks, mean = 0.01311, two-sided one-sample *t* test, *t* = 1.502, *df* = 35, *p* = 0.1420. The mean *ARI* between spatial and functional multilayer communities was near but significantly greater than zero (mean = 0.05364, one sample *t* test, *t* = 4.395, *df* = 22, *p* = 0.0002). This disagreement between spatial and functional partitioning was not driven by differences in community size, as *γ*, the spatial tuning parameter, was adjusted to minimize this difference (Figure 4H, paired *t* test, *t* = 0.1645, *df* = 22, *p* = 0.8709; see also Supporting Information Section F). This finding suggests that the functional connectivity of groups of neurons and astrocytes is largely independent of distance at this scale in vitro. Furthermore, the choice to use the multilayer modularity approach allowed us to calculate cell type specific participation across modules (Figure 4I). Relative to astrocyte segments, neurons participated in a greater fraction of functional (Tukey’s multiple comparisons test, *q* = 8.549, *df* = 88, *p* < 0.0001) and spatial (Tukey’s multiple comparisons test, *q* = 11.28, *df* = 88, *p* < 0.0001). This difference in module participation reflects the dominance of neurons in quantity, as subsampled networks with an equal number of neurons and astrocytes had near equal spatial and functional participation of both cell types (Supporting Information Section E, Figure S7E).

**Figure 5.**
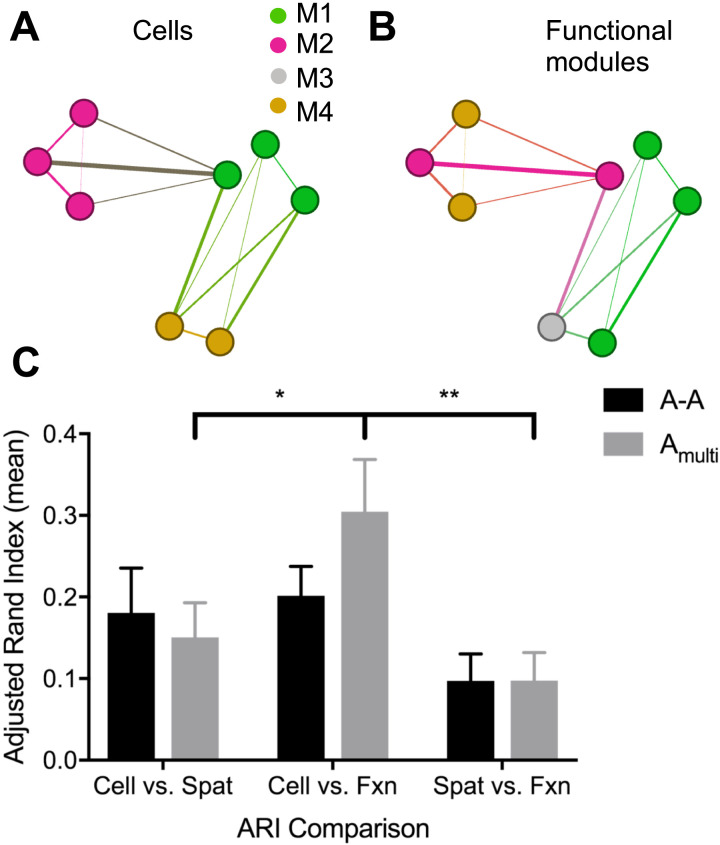
Functional and spatial communities of astrocyte segments are independent of cell membership, but less so in multilayer networks. (A) A representative astrocyte network with the indicated community structure based on morphology of segments. Nodes (astrocyte segments) of the same color belong to the same cell. (B) The network in panel A with modules detected based on functional connectivity. Nodes (astrocyte segments) of the same color belong to the same functional module, independent of cell membership. (C) Mean Adjusted Rand Index (*ARI*) for morphologically connected astrocyte segment communities (cell) versus spatially-connected communities, morphologically-connected versus functionally-connected segment communities, and functionally-connected versus spatially-connected communities. Morphological versus spatial *ARI* is larger than morphological versus functional, and also than functional versus spatial *ARI*s in both independent astrocyte (black) and multilayer (gray) astrocyte modules (two-way ANOVA comparing the effect of module type and single- or multilayer network on *ARI*: *F*(1, 132) = 7.675, *p* = 0.0062). The *ARI* is always larger for astrocyte communities generated using a multilayer network, though the improvement in *ARI* is not statistically significant (two-way ANOVA comparing the effect of single versus multilayer network type on *ARI*: *F*(2, 132) = 2.911, *p* = 0.0579). Error bars indicate standard error of the mean (SEM) and asterisks indicate statistical significance (*p ≤ 0.05, **p ≤ 0.01, ***p ≤ 0.001, ****p ≤ 0.0001).

Because astrocytes were subdivided into smaller functional units of microdomains, their functional modularity could be directly compared to their morphological (actual) modularity, which serves to group segments of the same cell (Figure 5A–B). This approach enabled us to test whether microdomains of the same astrocyte are functionally independent at the mesoscale. To test whether functional and spatial graphs of astrocyte segments matched actual cell morphology, we computed the *ARI* between functional, spatial, and actual astrocyte communities. Astrocyte microdomains were manually grouped into whole cells based on maximum fluorescence projection images (Supporting Information Figure S4). Community partitions between the three graph types (functional, spatial, and actual) were different, with *ARI* < 0.4 for all (Figure 5C). Interestingly, the *ARI* between cellular and functional communities was largest, significantly larger than cellular versus spatial (Tukey’s multiple comparisons test, *q* = 3.369, *df* = 132, *p* = 0.0486) and spatial versus functional (Tukey’s multiple comparisons test, *q* = 4.531, *df* = 132, *p* = 0.0048) community partitions generated from multilayer networks. Differences in community membership were not predominantly attributable to changes in community size or community number between graph types, as *γ* was tuned to minimize differences in mean community size of the three partitions at baseline (one-way ANOVA, *F*(2, 66) = 0.08331, *p* = 0.9202). Differences in actual, spatial, and functional modularity suggest that astrocyte segments behave relatively independently from the rest of the cell, and tend to cofunction with neurons.

When multi- and single-layer astrocyte *ARI*’s were grouped, differences between module types were significant (two-way ANOVA comparing the effect of module type and single or multilayer network on *ARI*: *F*(1, 132) = 7.675, *p* = 0.0062). This finding highlights the functional independence of astrocyte segments of the same cell in both their own layer and in the multilayer network. Further, *ARI* between all module types was improved when astrocyte communities were assigned from multilayer graphs, although this improvement was not statistically significant (two-way ANOVA comparing the effect of single versus multilayer network type on *ARI*, *F*(2, 132) = 5.840, *p* = 0.0037). That is, incorporating information about neural activity improved the agreement between morphologically and functionally determined astrocyte segment modules. One interpretation of this finding is that functional connectivity between astrocyte segments and neurons is more influenced by spatial proximity than is functional connectivity between astrocyte segments. Furthermore, this improvement in morphology-function agreement in multilayer astrocyte communities, which include neurons, suggests that the two populations are not functionally independent. If they were independent, we would expect the community structure of astrocyte segments to remain unchanged after multilayer modularity analysis.

## DISCUSSION

Using an in vitro model of traumatic brain injury, this study examined the functional response of cultured neuron-astrocyte populations to disruption of mGluR_5_-mediated signaling and mechanical injury. mGluR_5_ inhibition decreased neuronal, but not astrocytic, activity following injury, but did not alter higher order topological properties of multilayer networks on its own. In contrast, mechanical injury to neurons increased the global efficiency and clustering of the neuronal layer and clustering of the entire multilayer network, but did not affect the topology of the astrocyte layer. Pretreatment of injured cultures with an mGluR_5_ inhibitor attenuated the effect of injury on neuronal and multilayer topology.

In our mixed cultures, although neurons formed local spatial clusters, they functioned as a largely coupled network that did not depend on distance. In comparison, astrocyte domains formed a broad network with the potential for local “hot spots,” likely because one astrocyte has several astrocyte domains that we tracked separately. Our data show that both mechanical injury and mGluR_5_ signaling can affect the cohesiveness of mixed neuron-astrocyte networks, converting the neuronal network into a more localized network. Intriguingly, mGluR_5_ blockade attenuates the effect of injury on neuronal networks: neurons no longer increase their local connection strength after injury. In contrast, there is some effect of mGluR_5_ blockade alone on astrocyte-astrocyte networks, but no meaningful difference between injured, drug-treated networks and drug-treated networks.

The type I metabotropic glutamate receptors can shape synaptic strength, offering paths to both strengthen and weaken synapses in response to different input stimulation patterns (Neyman & Manahan-Vaughan, 2008). Specifically, activating mGluR_5_ facilitates effects on plasticity (Collingridge, Peineau, Howland, & Wang, 2010) that are almost independent of NMDAR-dependent plasticity (Thomazeau et al., 2021). Eliminating the injury-induced increases in local neuronal connectivity by blocking mGluR_5_ receptors suggests that activity patterns after our injury model favor local synaptic strengthening. Past work by our group shows that mechanical injury to entire populations, and not selected neurons, also leads to an enhancement of local connectivity for some in the population, but these changes were NMDA receptor dependent (Patel et al., 2014). However, others showed that mild mechanical injury to mixed cultures of neurons and astrocytes will also enhance NMDAR currents within individual neurons, presumably through the stretch activation of these receptors (Singh et al., 2012; L. Zhang, Rzigalinski, Ellis, & Satin, 1996), and these changes could be eliminated with MPEP treatment (Lea IV, Custer, Vicini, & Faden, 2002). These results imply that both the activity and scale of trauma can activate different mechanisms for remodeling the network. In either case, though, a consistent feature of injury is that neurons tend to form stronger localized connections during the repair process.

The same class of metabotropic glutamate receptors can also act on astrocytes and regulate gliotransmission. Activating mGluR_5_ on astrocytes has been shown to trigger glutamate release from astrocytes (Araque, Sanzgiri, Parpura, & Haydon, 1998; Parpura et al., 1994) and activate extrasynaptic NMDARs on neurons (Fellin et al., 2004). If this activation of extrasynaptic NMDARs if excessive, neuronal loss can occur (Ding et al., 2007; Dong, He, & Chai, 2013). Alternatively, modest activation of extrasynaptic NMDARs may influence some forms of LTP induction (Yang et al., 2017). Moreover, activation of mGluR_5_ on astrocytes can desynchronize neuronal networks, provided that astrocytes form a coordinated network of activity (Sasaki, Kuga, Namiki, Matsuki, & Ikegaya, 2011). One alterative explanation of mGluR_5_ mediating connectivity changes after mechanical trauma is that, in untreated and injured cultures, astrocytes respond (through the activation of mGluR_5_) by releasing glutamate, activating extrasynaptic NMDARs, and affecting the threshold for plasticity-induced remodeling of the circuit. In this case, blocking mGluR_5_ with MPEP will block the plasticity effect, explaining why MPEP blocks the injury-induced enhancement of connectivity. However, this effect may be mitigated by the role of mGluR_5_ in mediating neuronal synchrony; blocking mGluR_5_ may tip the circuit toward an activity pattern which would not favor local synaptic strengthening. Further studies are required to isolate the precise mechanism.

### Disrupted Neuronal Functional Connectivity Following Mechanical Injury, Modulated by mGluR_5_ Blockade

Previous studies using other in vitro models have shown that mechanical injury to neurons reduces functional connectivity (W. H. Kang et al., 2015; Myer et al., 2006; Patel et al., 2012). In line with these studies, our results show that single-cell tap injury to multiple neurons alters network topology in favor of increased clustering and larger global efficiency in the neuronal layer. Certainly, the precise removal of individual neurons from a network would decrease the synaptic inputs to neighboring neurons, leading to an acute reduction in the activity of the network. However, this could be followed by a robust homeostatic rescaling of network (Turrigiano & Nelson, 2004) which could subsequently alter the functional connectivity of the remaining neurons. Alternatively, randomly removing inhibitory neurons within the network, with their more restricted range of spatial connections, may also reorganize the activity to establish bursts of activity within smaller subnetworks, resulting in a network with an increased number of hubs, an increased efficiency, and an increase in clustering. Regardless of its exact origin, our results demonstrate the intricate interruption in balance across neuronal, but not astrocytic, layers that occurs from microtrauma to neurons.

Our observation that injured networks pretreated with MPEP did not exhibit the changes in topology observed for injured networks suggests that the mGluR_5_ receptor facilitates the neuronal response to injury. Specifically, the increased mean nodal strength, clustering, and global efficiency of neuronal layers following injury is likely to be facilitated, at least in part, by glutamatergic signaling via mGluR_5_. Because we do not observe these changes in the astrocyte layer or in interlayer strength, we speculate that neuronal mGluR_5_ is likely responsible. This finding is in agreement with previous work, which has found that long-term potentiation (LTP) at thalamic input synapses was impaired following bath application of MPEP in vitro (Rodrigues, Bauer, Farb, Schafe, & LeDoux, 2002). In in vivo follow-up, administration of MPEP dose-dependently impaired the acquisition of auditory and contextual fear learning, implicating mGluR_5_ in neuronal plasticity, and highlighting the functional importance of this receptor. In another study, mGluR_5_-deficient mutant mice showed a complete loss of the NMDA-receptor mediated component of LTP in hippocampal CA1 neurons (Jia et al., 1998). From these and our results, we infer that mGluR_5_ plays an important role in establishing and maintaining the functional connectivity of neural circuits.

While injury did not alter astrocyte population activity, it did change the topology of multilayer networks in a manner that was not observed completely in neuron or astrocyte populations alone. Specifically, injury increased mean nodal strength, clustering, and global efficiency in the neuronal layer, but did not significantly affect mean strength of multilayer, interlayer, or astrocyte layer nodes. However, injury did increase the clustering, but not global efficiency, of multilayer networks. Taken together, these results suggest that multilayer changes in clustering coefficient are mostly mediated by the neuronal layer.

Conceptually, increased mean nodal strength in the neuronal layer following injury may result from local changes in synaptic strength from plasticity, or may also result from NMDA receptor subtypes influencing the remodeling of synaptic strength. On its own, spike timing dependent plasticity can compensate quickly for reductions in activity that occur when a fraction of the neuronal population is inactivated, leading to a recovery in connectivity within the microcircuit and an increase in remaining synaptic strength (Gabrieli, Schumm, Vigilante, Parvesse, & Meaney, 2020; Schumm, Gabrieli, & Meaney, 2020), in turn increasing the functional connectivity in the network. Our model of mechanical trauma may lead to local increases in functional connectivity within neurons containing a large fraction of NMDAR receptors containing the GluN_2_A subunit (Patel et al., 2014). Injury also increased the clustering coefficient, reflective of closure in local neighborhoods in the multilayer network. These topological changes may result from functionally eliminating nodes through which long-distance paths pass, forcing more of the remaining healthy neuronal nodes to adopt hub roles, thereby increasing local clustering. In this light, the elimination of nodes appears to promote a rerouting information through other healthy nodes to indirectly trigger a ‘pathological plasticity,’ where increases in functional connectivity are a necessary step in rebuilding the network. The interaction of injury and drug did not have this effect on clustering, suggesting that mGluR_5_ inhibition prevents the impact of injury on topology. This protective effect of mGluR_5_ inhibition following injury was observed in previous studies in vitro and in vivo (Lea et al., 2002; Movsesyan et al., 2001), and has been attributed to a reduction in NMDAR activity following mGluR_5_ inactivation.

### Unchanged Neuron-Astrocyte Connectivity Following mGluR_5_ Inhibition

While molecular and cellular changes following direct injury to astrocytes is well-documented (Charles et al., 1991; Ellis et al., 1995; Rzigalinski et al., 1998), the functional contribution of astrocytes to neural population activity in response to targeted neuronal injury is unknown. Due to the expression of mGluR_5_ on neurons and astrocytes, we were unable to target astrocyte-specific mGluR_5_ pharmacologically. Thus, we could not determine whether changes in neuronal network density were attributable to changes in gliotransmission. Contrary to our initial hypothesis, astrocyte activity and functional connectivity did not decrease following injury or mGluR_5_ inhibition. Because we did not directly injure astrocytes, their lack of direct response to injury after 1 hour is perhaps expected.

However, the lack of astrocyte response to mGluR_5_ blockade is more difficult to interpret. It could be that astrocytes are able to compensate for impaired glutamatergic signaling through other modes of intra-astrocyte communication, including gap junctions and other biochemical receptor pathways. Alternatively, we may have examined astrocyte activity at too coarse of a spatial scale. Astrocyte mGluRs are localized in microdomains, which we were unable to visualize under cytosolic GCaMP6f or GFAP staining. Thus, we likely did not observe the full extent of astrocyte activity and its dynamics following injury and mGluR_5_ inhibition. Improper spatial resolution might also explain the low expression of mGluR_5_ on astrocytes relative to neurons observed in stained cultures. Additionally, previous work indicating a critical role for mGluR_5_-mediated neuron-astrocyte communication was primarily conducted in slices and in vivo, rather than in dissociated cultures. Thus, it is possible that different gliotransmission pathways, perhaps purinergic (Panatier & Robitaille, 2016), are relatively more important in our cultures, which differ in age, health, cellular composition, and genetic variation from astrocytes in slices and in vivo.

### Independence of Spatially and Functionally Predicted Community Structure in Small-Scale Neuron-Astrocyte Networks

Although significant progress has been made toward understanding spatially embedded networks and community structure in vivo (Bassett & Stiso, 2018; Betzel & Bassett, 2017), functionally and structurally informed modularity of in vitro neuronal networks has not been explored. Because cultured cells exist in a constrained 2-D environment, devoid of connective tissue and vasculature, the spatial organization of in vitro networks is likely to be different from that of in vivo networks. Furthermore, we examined a small subsection of a larger network in this study. In this context, it is logical that the functional and spatial community structure of neuron-astrocyte networks are mostly independent. Neurons and astrocytes can connect with one another over distances an order of magnitude larger than the approximately 1-mm field of view imaged in this study. Thus, functional connectivity can be established largely independent of spatial proximity in healthy networks. It could be that spatial proximity strongly influences functional modularity at and above a certain scale, or when the network is under stress.

Comparing spatial and functional network modularity in astrocyte segments facilitated more general characterization of astrocyte calcium signaling. Astrocytic calcium events are heterogeneous in temporal and spatial profile, ranging from fast, locally restricted events to slower, cell-wide waves (Bazargani & Attwell, 2016; Stobart et al., 2018; Y. Wang et al., 2018), with these two types of events often occurring independently (Di Castro et al., 2011; Panatier et al., 2011). There is limited understanding of how Ca^2+^ fluctuations in microdomains relate to cell-wide, or population-wide, astrocyte calcium events (Nimmerjahn & Bergles, 2015). Analyzing astrocyte segments as nodes in a spatially embedded network enabled us to elucidate the extent to which segments of the same astrocyte are functionally interdependent. If segments of the same astrocyte participate in different functional modules, there is likely a functional advantage to maintaining strong interastrocyte and astrocyte-neuron connectivity above intra-astrocyte connectivity. This microdomain independence allows a given astrocyte to sense and integrate activity from a diverse set of neurons over time and space (Araque et al., 2014; Fields, Woo, & Basser, 2015). Multilayer astrocyte modularity recapitulated this mesoscale structure-function independence, but to a lesser degree.

### Methodological Considerations

A major limitation of this study is the extreme sparseness of active astrocyte segments relative to neurons in our cell cultures. Neuronal dominance of multilayer network dynamics likely skewed multilayer topology toward the neuronal layer. The low level of activity in astrocytes could be due to our examination of astrocyte activity at specific spatial and temporal scales or to the health of the cells in culture. Prior work indicating critical roles for neuron-astrocyte communication in plasticity, synchronization, and behavior were primarily conducted in slices and in vivo, and many involved direct stimulation of neurons or promoted arousal to evoke an astrocyte response (Srinivasan et al., 2015; X. Wang et al., 2006). In contrast, we examined relatively asynchronous spontaneous neural activity in dissociated cultures. The astrocytes in our model system may not have developed the complete range of neuromodulatory activities performed by those in vivo.

The interpretation of our findings depends on relative mGluR_5_ expression and signaling importance in the neurons and astrocytes in our cultures. The presence and importance of mGluR_5_ on neurons and in their signaling is well documented (Ballester-Rosado et al., 2010; Gass & Olive, 2009; Shigemoto et al., 1993) and has been confirmed with immunofluorescent staining of mGluR_5_ in our in vitro preparations. Indeed, the decrease in neuronal network density following MPEP administration is likely attributable to blockade of neuron-neuron communication. This is an inherent limitation in conventional pharmacological studies of gliotransmission. To isolate the causal effect of astrocyte-specific signaling on neural circuit activity, future experiments could use more sophisticated tools such as designer receptors exclusively activated by designer drugs (DREADDs) (Scofield et al., 2015), astrocyte-specific G-protein coupled receptor opsins (Mederos et al., 2019), or genetic knockdown (Shigetomi, Tong, Kwan, Corey, & Khakh, 2012).

### Future Directions: In Vivo Network Models of Neuron-Astrocyte Signaling and Expanded Models of Neuron-Glia Networks

The aforementioned differences in health and activity between astrocytes in vitro and in vivo motivate future work comparing the topology and response behavior of neuron-astrocyte networks in cell culture and animal models. Assuming that the number of active astrocyte microdomains in vivo is greater than in culture, we speculate that there would be significant changes in neuron-astrocyte network community structure, as astrocytic participation in spatial and functional models would increase. Similarly, we speculate that there is increased density and strength of astrocyte-astrocyte and neuron-astrocyte connections in vivo. Given our findings that betweenness centrality, global efficiency, and clustering are mostly influenced by density and strength, these topological properties would change accordingly in in vivo networks. Furthermore, with greater neuron-astrocyte connectivity in vivo, neuronal manipulations, such as injury, would induce a greater change in multilayer and astrocyte layer network topology. Additionally, we propose that the topology of neuron-astrocyte networks changes from its spontaneous state during periods of intense stimulation, given that astrocytes exhibit unique responses to stimulation and during behavior (Halassa et al., 2009; Lee et al., 2014; Perea, Yang, Boyden, & Sur, 2014; Schummers, Yu, & Sur, 2008; Srinivasan et al., 2015; X. Wang et al., 2006). For example, there may be increased alignment between morphological and functional astrocyte communities during stimulation, as repeated neuronal firing triggers global astrocyte Ca^2+^ events (Khakh & Sofroniew, 2015). Indeed, astrocytes in vivo, which occupy non-overlapping territories, have been hypothesized to form functional “islands” of neuronal synapses (Halassa, Fellin, Takano, Dong, & Haydon, 2007). It is difficult to speculate how our findings related to mGluR_5_ inhibition will translate in vivo. The relevance of mGluR_5_-mediated gliotransmission in adult rats is debated, as there is evidence of decreased mGluR_5_ expression after 7–21 days postnatally (Cai, Schools, & Kimelberg, 2000; Morel, Higashimori, Tolman, & Yang, 2014; Sun et al., 2013).

The field of network neuroscience was largely built upon observations of structural connectivity at the whole brain or macroscale represented as graphs (Bassett & Bullmore, 2017; Bassett et al., 2018; Liao, Vasilakos, & He, 2017). Combined with the ever-increasing focus on data science, the field has left first-principles descriptions of functional connectivity at the elementary (single-neuron) level relatively underexplored (Bassett et al., 2018) Indeed, there have been few functionally-derived network models of neuronal microcircuits (Bettencourt, Stephens, Ham, & Gross, 2007; Bonifazi et al., 2009; W. H. Kang et al., 2015; Patel et al., 2012; Schroeter, Charlesworth, Kitzbichler, Paulsen, & Bullmore, 2015; Srinivas, Jain, Saurav, & Sikdar, 2007). Our work aids in addressing this understudied area by characterizing the topology, and changes therein, of functional connections between elementary-scale mixed cortical cultures. Further, to our knowledge, the field of network neuroscience is devoid of any models describing or predicting connectivity between neurons and glia, although there are some computational neuroscience models that explore the interactions of these two cell types. Network modeling of neuron-glia interactions is an area ripe for future study, and is particularly well suited for characterization and prediction using multilayer models.

The field of network neuroscience has used multilayer networks to model brain network development over time, to compare healthy and diseased brains, to predict structure–function relationships, and to link multiscale and multimodal data (Bassett et al., 2011; Betzel & Bassett, 2017; Betzel et al., 2018; Muldoon & Bassett, 2016; Pedersen, Zalesky, Omidvarnia, & Jackson, 2018). Despite the increasing popularity of multilayer networks and the existence of multiple neural cell types, there has been limited use of the multilayer framework to study interactions between two or more cell populations. Here, we demonstrate the validity of a multilayer network model to characterize the connectivity of neuron-astrocyte populations.

### Conclusion

Our results indicate that mGluR_5_ is not essential for maintaining spontaneous astrocyte-astrocyte or neuron-astrocyte functional connectivity in noninjured cultures. However, mGluR_5_ blockade partially restored baseline topology of multilayer networks following traumatic injury, underscoring the potential therapeutic benefit of modulating glutamatergic tone. More broadly, we have proposed and demonstrated the utility of a multilayer network model of in vitro neuron-astrocyte populations in interpreting the topological impacts of various experimental manipulations. Our findings support the notion that neurons and astrocytes are not functionally distinct populations, but rather display an interdependent topology that reveals network responses not observed in either population alone. Future work could aim to isolate the astrocytic contribution to static and dynamic network topology using more targeted experimental manipulations, and could study healthy neuron-astrocyte populations in vivo, where astrocytes may be more abundant and active.

## MATERIALS AND METHODS

### Experimental Protocol

All animal procedures were approved by the University of Pennsylvania Institutional Animal Care and User Committee. Timed pregnant Sprague-Dawley rats were anesthetized with 5% CO_2_ and sacrificed via cervical dislocation. Embryos at day E18 were surgically removed and their neocortical tissue dissected and dissociated for 15 min at 37°C in trypsin and DNAse. Following trituration and filtration through Nitex mesh, cells were resuspended in minimum essential media (MEM) with Earl’s salts and GlutaMAX supplemented with 0.6% D-glucose, 1% Pen-Strep, and 10% Horse Serum. Cells were plated on glass-bottomed Matteck dishes coated in poly-D-lysine and laminin at a density of 300,000 cells/mL. Cultures were grown in a humidified incubator at 37°C and 5% CO_2_, maintained in serum-free MEM and 0.2% GlutaMAX (Gibco), and fed twice weekly with 1X NEUROBASAL medium (ThermoFisher).

To visualize the calcium activity of astrocytes in addition to neurons, cells were transduced using Adeno-associated virus Serotype 1 (AAV1) carrying the gene for GCaMP6f under a CAG promoter, which is general to mammalian cells (AAV1.CAG.Flex.GCaMP6f.WPRE.SV40, Addgene), at DIV 3. All manipulations and imaging were performed at DIV 10. Dishes were randomly chosen to be treated with 1 uM 2-Methyl-6-(phenylethynyl)pyridine (MPEP), a potent mGluR_5_ inhibitor (for 19 dishes), or MEM only (*n* = 36 dishes total, Supporting Information Figure S10). Calcium images were obtained at 488 nm excitation, 50 ms exposure on a Nikon Eclipse TE2000 confocal microscope. Eight to nine dishes in each treatment group were mechanically injured by briefly deforming the cell surface of 12–15 neurons with a vertical movement of a pulled glass micropipette tip (Charles et al., 1991). The micropipette was controlled manually with a microcontroller (Eppendorf), directly perturbing the cell with the pipette tip. Care was taken to avoid rupturing the cell membrane, which would have caused immediate cell death. This model enabled precise, controlled injury of a similar severity across dishes.

### Image Processing

Images were manually segmented to identify neurons and astrocyte microdomains as regions of interest (ROIs, Supporting Information Figure S9, Figure S4). Neuronal and astrocytic fluorescence traces were extracted from each ROI as pixel intensity over time (Supporting Information Figure S9). Neuronal and astrocytic events were identified automatically, using a custom-built MATLAB template-matching algorithm (Patel et al., 2015), based on the trace’s correlation with a library of true neuron and astrocyte calcium waveforms (Supporting Information Figure S11, Figure S12). Automatically identified events were manually confirmed based on calcium traces or eliminated following event detection. Because astrocytic activity was mostly localized to processes that often fluoresced independently, an observation that has been confirmed in previous studies (Fields et al., 2015; Srinivasan et al., 2015), astrocyte segments were analyzed as separate entities. Cells predicted to be astrocytes based on morphology were confirmed based on their functional response to 100-uM NMDA administered with 1 uM of glycine co-agonist (Supporting Information Section B). Only ROIs that had one or more confirmed calcium events in one or more recordings were kept for further analysis. This pruning step eliminated a significant number of nonactive astrocyte segments, reducing the number of dishes with two or more active astrocyte segments to 23 from 36.

### Generation of Adjacency Matrices

We based functional connectivity on filtered, scaled calcium fluorescence. For a pair of ROIs, either neuronal or from an astrocyte microdomain, we calculated the Pearson’s correlation coefficient between the filtered, scaled calcium fluorescence trace and 100 surrogates with similar properties to the original traces. Specifically, the surrogate time series had the same marginal distribution and power spectrum as the original, and generated using an amplitude adjusted Fourier transform (AAFT) algorithm (Kugiumtzis & Tsimpiris, 2010). Only pairs of ROIs that had a phase difference significantly larger or smaller than the surrogates, as determined by a *z* test with *p* < 0.05, were linked with an edge having a weight equal to the correlation between the two traces (Supporting Information Section C). Multilayer adjacency matrices were organized as shown in Figure 2, with neuron-neuron, neuron-astrocyte, and astrocyte-astrocyte adjacency matrices concatenated. Spatial adjacency matrices were generated by measuring the radial distance between the center of each ROI of each node pair, (*x*1, *y*1) and (*x*2, *y*2), as √(|(*x*2 − *x*1)|^2^ + |(*y*2 − *y*1)|^2^). The distance was normalized to the maximum possible distance in the field of view, and subtracted from one so that the pairs of nodes with the shortest radial distance were linked by edges with the highest weights. Null networks were generated by randomizing adjacency matrices while preserving degree distribution and density using the *randmio* function in the BCT (Maslov & Sneppen, 2002). For multilayer networks, randonization was perfomred on the entire adjacency matrix as a whole, rather than randomizing neuron and astrocyte layers separately. Edges in networks with more than three edges were rewired 100 times for neuron networks, 10 times for astrocyte networks due to their smaller size, and 100 times for multilayer networks.

### Analysis of Relationship Between Spatial and Functional Connectivity

To examine the degree to which functional connectivity depends on spatial connectivity, we regressed functional edge weight against spatial edge weight, pooling weights across networks of the same experimental condition at the final experimental time point. In addition, we calculated the 2-D correlation coefficient between spatial and functional adjacency matrices (using MATLAB’s *corr2* function) for all dishes pooled at baseline. To account for spurious correlation, we subtracted from this value the 2-D correlation coefficient between two random matrices of the same size with weights randomly sampled from a uniform distribution. To investigate whether the probability of a functional edge existing at all (e.g., connection probability) depends on spatial connectivity, we grouped functional edges into 30 bins based on their spatial connection weight using MATLAB’s *histcounts* function. The functional connection probability was calculated as the number of nonzero edges in the bin divided by the total number of edges in the bin. We then regressed functional connection probability against the mean spatial connection weight in the bin. Edges were pooled across dishes of each condition at the third experimental time point.

### Calculation of Network Statistics

Density, strength, clustering coefficient (*C*), betweenness centrality (*B*), and global efficiency (*E*) were calculated for neuron, astrocyte, and multilayer networks by using weighted adjacency matrices. Equations are provided in the Supporting Information Section D. In most cases, MATLAB scripts from the BCT were used for calculations (Rubinov & Sporns, 2010). The form of the multilayer adjacency matrices lends itself to straightforward calculation of network statistics in the same way as for single-layer networks. To compare the topology of the two layers and intra- versus interlayer connections, we calculated mean nodal strength of the entire network, mean nodal strength of each individual layer, and mean strength of interlayer connections. Interlayer strength and density were calculated by summing the weights, or number of nonzero values, of the edges between neurons and astrocytes. For a node in a given layer, interlayer nodal strength was normalized by the number of nodes in the other layer, for example (*N*_*n*_ for nodes in the astrocyte layer, where *N*_*n*_ is the number of neurons). More global network statistics, such as betweenness centrality, global efficiency, and clustering coefficient, cannot be separated into intra- and interlayer components. These metrics were calculated for the entire multilayer network and also for individual layers. To estimate the effect of treatment group assignment on these graph statistics (*B*, *C*, and *E*), we ran a generalized linear regression (Nelder & Wedderburn, 1972) using the GLM module class in Python’s *statsmodels* module (Seabold & Perktold, 2010). The link function was the identity and error distribution was assumed to be normal. Outcome variables *B*, *C*, and *E* were regressed against categorical variables indicating treatment group (MEM, No Injury (Sham); MPEP, No Injury (Sham); MEM, Injury; or MPEP, Injury), as well as mean nodal strength, to control for its effects on topological statistics. Regression coefficient estimates and *p* values are reported above.

### Community Detection in Spatial and Functional Graphs

To better understand how individual astrocyte segments and neurons give rise to collective network structure, we analyzed the multilayer network’s mesoscale community structure (Betzel & Bassett, 2017; M. A. Porter, Onnela, & Mucha, 2009; Supporting Information Figure S13). Modules are communities of nodes that are functionally or spatially more interconnected to each other than to other nodes in the network. Modules were generated by maximizing the modularity quality function using both spatial and functional adjacency matrices for single-layer neuron and astrocyte networks, and for multilayer networks. The parameter *γ* is a resolution parameter that governs the size and number of detected communities. For both single-layer and multilayer networks, *γ* was tuned separately and manually for spatially and functionally generated graphs to minimize the difference between the number of communities detected for spatial and functional networks (Supporting Information Section F). Quality was maximized by placing nodes into communities such that the quality *Q* is as large as possible, using an iterative Louvain-like community detection algorithm implemented in MATLAB (Jutla et al., 2011). A Girvan-Newman (Newman & Girvan, 2004) null model was used for both functional and spatial graphs and implemented in a custom MATLAB script (Supporting Information Section F). Because the modularity landscape is rough (Good, De Montjoye, & Clauset, 2010), the heuristic can detect a different partition each time it is applied. Thus, here we assign a node’s module to be the mode over 50 maximizations. Nevertheless, module assignment still varies slightly between batches of 50 trials, a property that more generally has been demonstrated for small networks (Good et al., 2010). In addition to the value of *Q*, the Louvain algorithm outputs the partition *g*, a vector containing the community number of each node.

We compared the similarity of the partitions predicted by functional and structural graphs by calculating their *ARI* (Hubert & Arabie, 1985; Rand, 1971) using a MATLAB algorithm (Randindex, 2000). The Rand Index represents the probability that the two partitions, functional and spatial, will agree on the community of a randomly chosen node. A Rand Index of zero indicates that the two partition vectors do not have any groupings in common, while a value of one indicates complete agreement. The *ARI* corrects for overinflation due to chance assignment (Supporting Information Section F). Module participation for each cell type was calculated as the fraction of all modules that contain at least one of that cell type.

### Statistical Testing

Two-sided one-sample *t* tests were conducted to determine the statistical significance of the difference between actual and null network statistics, as well as the statistical significance of the difference of the mean *ARI* from zero for neuron layer (*n* = 36 dishes), astrocyte layer (*n* = 23 dishes) and multilayer (*n* = 27 dishes) networks. Two-sided paired *t* tests were conducted to determine whether there existed a statistically significant difference between the size of functionally and spatially determined modules for neuron (*n* = 36 dishes), astrocyte (*n* = 23 dishes), and multilayer (*n* = 27 dishes) networks. A two-sided unpaired *t* test was used to test for a statistically significant difference between the size of multilayer network functional communities in MPEP- and MEM-treated dishes. One-way analysis of variance (ANOVA) was used to test for a significant difference between means of a single measure between multiple groups. Two-way ANOVA was used to test for a significant difference between means of multiple measures between multiple groups. The Holm-Sidak method was used to correct for multiple comparisons when making post-ANOVA comparisons between the means of selected pairs of groups. The Tukey method was used to correct for multiple comparisons when making post-ANOVA comparisons between every mean and every other mean. Two-sided *t* tests were used to test for statistical significance of all regression coefficients.

## ACKNOWLEDGMENTS

We thank S. Schumm, K. Wiley, and P. Srivastava for helpful comments on an earlier version of this manuscript; A. Nam for primary cell culture; and O. Teter for collaborative brainstorming.

## SUPPORTING INFORMATION

Supporting information for this article is available at https://doi.org/10.1162/netn_a_00227.

## AUTHOR CONTRIBUTIONS

Margaret Schroeder: Conceptualization; Data curation; Formal analysis; Funding acquisition; Investigation; Methodology; Project administration; Software; Validation; Visualization; Writing – original draft; Writing – review & editing. David Meaney: Conceptualization; Funding acquisition; Investigation; Methodology; Project administration; Resources; Supervision; Writing – review & editing. Danielle Bassett: Conceptualization; Investigation; Methodology; Project administration; Supervision; Writing – review & editing.

## FUNDING INFORMATION

David Meaney, Paul G. Allen Frontiers Group, Award ID: 12347. Danielle Bassett, Allen Foundation (https://dx.doi.org/10.13039/100008796). David Meaney, National Institute of Neurological Disorders and Stroke (https://dx.doi.org/10.13039/100000065), Award ID: 1RO1-NS 088276. Danielle Bassett, John D. and Catherine T. MacArthur Foundation (https://dx.doi.org/10.13039/100000870). Danielle Bassett, Alfred P. Sloan Foundation (https://dx.doi.org/10.13039/100000879). Danielle Bassett, ISM Foundation. Danielle Bassett, Army Research Laboratory (https://dx.doi.org/10.13039/100006754), Award ID: W911NF-10-2-0022. Danielle Bassett, Army Research Office, Award ID: Bassett-W911NF-14-1-0679, Grafton-W911NF-16-1-0474, DCIST-W911NF-17-2-0181. Danielle Bassett, Office of Naval Research. Margaret Schroeder, National Institute of Mental Health (https://dx.doi.org/10.13039/100000025), Award ID: 2-R01-DC-009209-11, R01-MH112847, R01-MH107235, R21-M MH-106799. Danielle Bassett, National Institute of Child Health and Human Development (https://dx.doi.org/10.13039/100000071), Award ID: 1R01HD086888-01. Danielle Bassett, National Institute of Neurological Disorders and Stroke (https://dx.doi.org/10.13039/100000065), Award ID: R01 NS099348. Danielle Bassett, National Science Foundation (https://dx.doi.org/10.13039/100000001), Award ID: BCS-1441502, BCS-1430087, NSF PHY-1554488, and BCS-1631550.

## TECHNICAL TERMS

Astrocytes: Star-shaped glial (nonneuronal) cells of the central nervous system responsible for a variety of functions.
Glutamate: A critical neurotransmitter at excitatory synapses and the most abundant free amino acid in the brain.
Gliotransmission: The calcium-dependent release of neurotransmitters, (including glutamate, D-serine, and ATP) from astrocytes that has been demonstrated in some preparations.
Traumatic brain injury: Physical, mechanical trauma to neural cells resulting from a blow or jolt to the head or body.
Multilayer networks: A more general network framework which allow for the interaction of multiple node types across layers of a single node type.
